# Modified femoral pressuriser generates a longer lasting high pressure during cement pressurisation

**DOI:** 10.1186/1749-799X-6-54

**Published:** 2011-10-17

**Authors:** Jian-Sheng Wang, Göran Garellick, Fred Kjellson, Elizabeth Tanner, Gunnar Flivik

**Affiliations:** 1 Department of Orthopedics, Clinical Sciences Lund, Lund University and Skåne University Hospital, Lund, S-221 85, Sweden; 2 Biomaterials and Biomechanics Unit, Lund University, Lund, S-22185, Sweden; 3 Department of Orthopaedics, Institute of Surgical Science, Sahlgrenska University Hospital, Göteborg University, Mölndal, Sweden; 4 School of Engineering, University of Glasgow, Glasgow, G12 8QQ, UK

**Keywords:** Pressuriser, pressurisation, THA and cementing technique

## Abstract

**Background:**

The strength of the cement-bone interface in hip arthroplasty is strongly related to cement penetration into the bone. A modified femoral pressuriser has been investigated, designed for closer fitting into the femoral opening to generate higher and more constant cement pressure compared to a commercial (conventional) design.

**Methods:**

Femoral cementation was performed in 10 Sawbones^® ^models, five using the modified pressuriser and five using a current commercial pressuriser as a control. Pressure during the cementation was recorded at the proximal and distal regions of the femoral implant. The peak pressure and the pressure-time curves were analysed by student's t-test and Two way ANOVA.

**Results:**

The modified pressuriser showed significantly and substantially longer durations at higher cementation pressures and slightly, although not statistically, higher peak pressures compared to the conventional pressuriser. The modified pressuriser also produced more controlled cement leakage.

**Conclusion:**

The modified pressuriser generates longer higher pressure durations in the femoral model. This design modification may enhance cement penetration into cancellous bone and could improve femoral cementation.

## Background

Since 1979 the Swedish Hip Arthroplasty Register has documented improvements in cementing techniques. Changes in the "Modern Cementing Technique" have been linked to at least 20% reduction in revision rates for aseptic loosening [1]. The strength of the cement-bone interface is strongly related to cement intrusion into the bone [2]. The shear strength of the cement-bone interface has been investigated since the 1970's. Studies showed that maximal cement-bone interface shear strength is related to thoroughly cleaned strong trabecular bone with a deep cement penetration [3]. Buckley et al. [4] showed that bone cement interdigitated into cancellous bone was better able to resist fracture than bone cement alone. The depth of cement intrusion correlates with the cement-intrusion pressure [5-10]. High and constant pressure, both to resist the force of blood pressure and to force the cement into the spaces in the cancellous bone, is necessary during cement filling. The purpose is to reach extensive micro-interlock [11-14]. Therefore, increasing cement pressurisation and duration is an essential component of cementing to ensure good cement-bone contact throughout the femoral cavity, radiographic "whitening out" and a stronger bone-cement interface.

The aim of a proximal femoral seal is to keep the femur closed while the cement is injected and thereby provide high cementation pressure resulting in better cement penetration. Some cement leakage always occurs from the proximal femur even when using a pressuriser and this leakage lowers the pressure generated in the femoral canal and thus cement penetration into cancellous bone [15]. As we found many existing commercial pressurizers allow too much cement leakage during pressurisation, we designed a modified femoral pressuriser, trying better to account for the anatomic proximal femoral contour. The new design was based on a combination of multiple measurements from templating total hip arthroplasties at our hospital and the involved surgeons' clinical experience. The aim was to improve the proximal seal and thus to achieve higher and more constant pressure in the femoral canal with reduced and more controlled leakage. This new design of pressuriser was investigated using Sawbones^® ^model proximal femora and the results compared to a conventional pressuriser.

## Materials and methods

**Figure 1:**
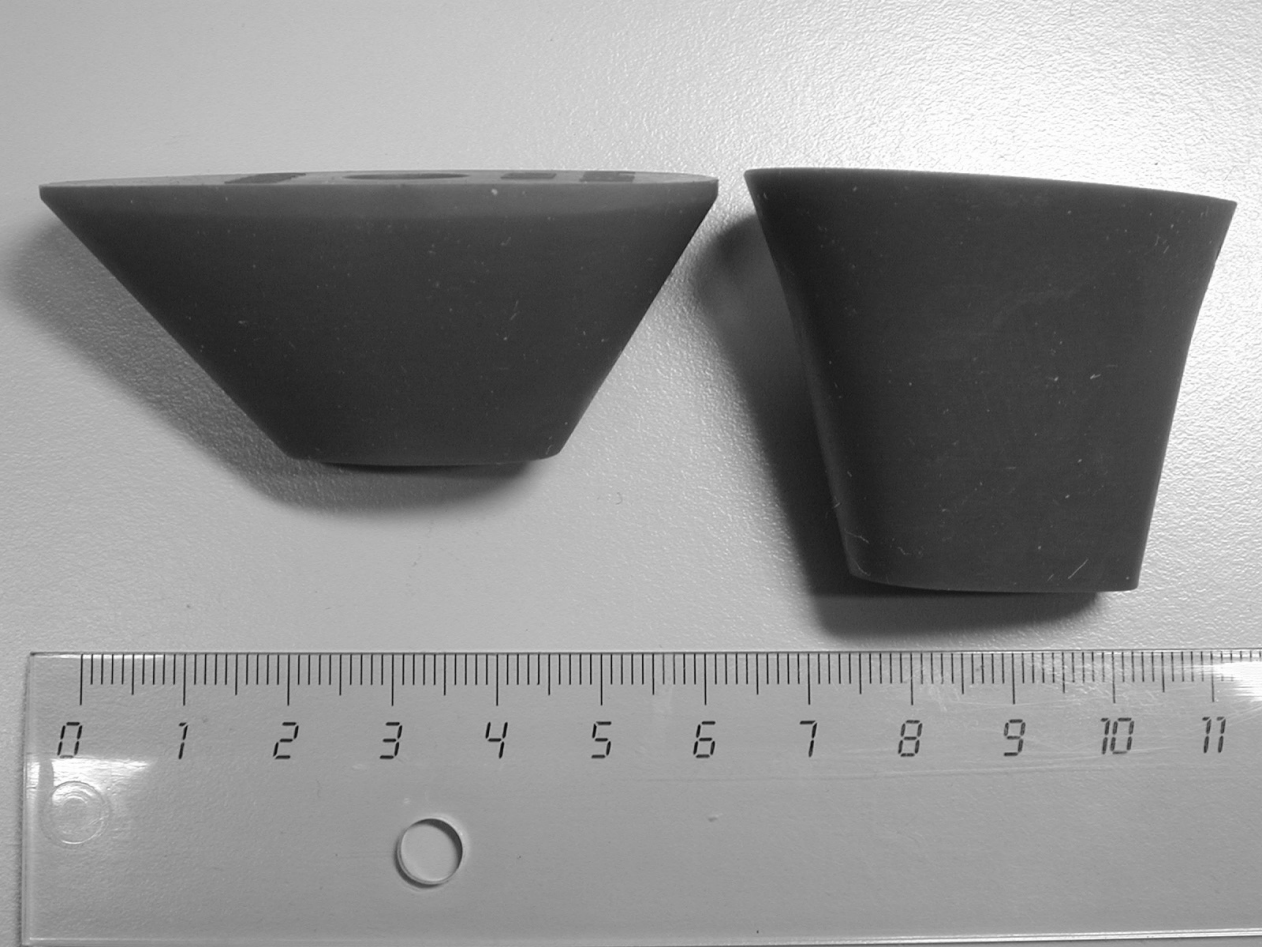
**Two types of cement pressurisers. Conventional pressuriser on left side and modified pressuriser on right side**.

**Figure 2:**
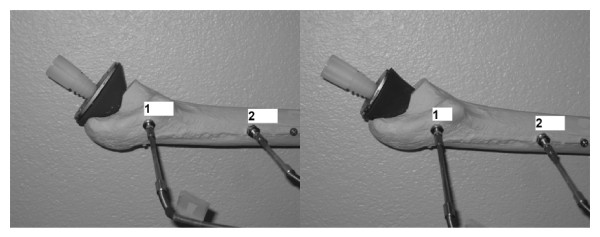
**The pressure transducers fixed within the Sawbone^® ^model proximal femora and showing the more gradual taper of the conventional design (left) compared to the modified design (right)**. With both pressurisers a newly developed metal backing plate was used to support the pressuriser.

The conventional pressuriser was provided by Biomet Cementing Technologies AB (Sjöbo, Sweden). The modified pressuriser was made longer in the proximal-distal direction and narrower in the anterior-posterior and medial-lateral directions with a narrower taper angle compared to the conventional design (Figure 1 and Table 1). The modified pressuriser was made of the same silicone material as the conventional and both designs were reinforced with a newly developed 2 mm thick steel backing plate (Figure 2) which aimed at assisting in load transfer from the cement gun.

**Table 1:** The geometrical differences between the two pressurisers.

Pressuriser	Proximal level		Distal level		Height
	***Width***	***Thickness***	***Width***	***Thickness***	

**Conventional**	62 mm	28 mm	25 mm	13 mm	26 mm

**Modified**	47 mm	31 mm	28 mm	15 mm	37 mm

Ten Sawbones^® ^proximal femora were prepared based on standard operative procedures. The femoral neck osteotomy was standardised to a cut at 20 mm from the lesser trochanter. The femoral canal was reamed to fit a Biomet Optima^® ^femoral prosthesis allowing for 3 mm cement mantle. A distal plug (Optiplug, Biomet Cementing Technologies AB) was inserted into the canal, to provide a 10 mm prosthesis distal tip to plug distance. To measure the cement pressure two modified Entran EPB-B02 pressure transducers (Entran, UK) were inserted and fixed into drill holes made on the lateral femoral shaft (Figure 2). The positions were standardised at 50 mm (position 1) and 140 mm (position 2) from the tip of the greater trochanter.

80 g of pre-chilled (4°C) Palacos R was mixed using an Optivac^® ^cement mixing system (Biomet Cementing Technologies AB, Sjöbo, Sweden) under vacuum (0.15 bar) in a temperature controlled room at 21 ± 1°C. At 2 minutes 15 seconds after start of mixing, the cement was injected in a retrograde manner into the Sawbones^® ^model bone using a cement gun (Optigun, Biomet Cementing Technologies AB, Sjöbo, Sweden) with a long nozzle. Immediately after the end of cement injection a pressuriser, reinforced with the metal backing plate, was placed on to the nozzle which was cut close to the end of the pressuriser. The cement gun was placed on the calcar opening on the proximal femur and load was applied from 2 min and 45 seconds to 4 minutes 30 seconds after the start of cement mixing using as standard manner as possible, with gradual injection of additional cement. The leakage of the cement was observed and the manner of leakage noted. Use of the conventional or modified pressuriser was alternated. In total five modified pressurisers, with five conventional ones as controls, were used in the study. The Sawbones^® ^preparation and subsequent pressurization procedure were performed by two experienced hip surgeons in cooperation (GG and GF).

The pressure-time curves were recorded throughout the cementation procedure. The peak pressure and time durations when different pressure thresholds were exceeded (100, 150, 200, 250 and 300 kPa) were analysed. Statistic analysis was performed using student's t-test for peak pressure and Two-way ANOVA for time over these pressure thresholds.

## Results

**Figure 3:**
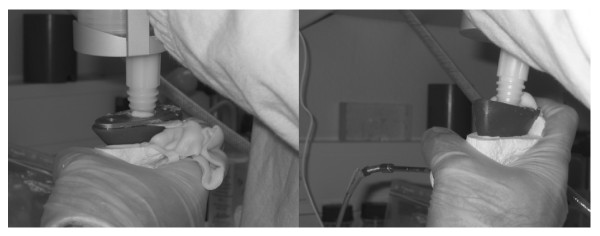
**During the pressurisation the cement leaked more around the conventional pressuriser (left) compared to the modified pressuriser (right)**.

**Figure 4:**
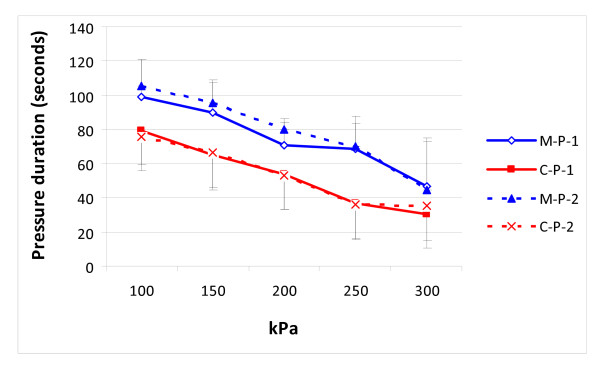
**The mean durations of the different pressures generated in the femoral canal with modified (blue lines) and conventional pressurisers (red lines) at positions 1 and 2**. M-P-1 = Modified position 1 - Proximal. M-P-2 = Modified position 2 - Distal. C-P-1 = Conventional position 1 - Proximal. C-P-2 = Conventional position 2 - Distal

Using the modified pressuriser the cement tended to leak from only one zone around the modified pressuriser (the notch in the lateral-posterior region) whereas leakage occurred from all zones around the conventional pressurisers (Figure 3). For the pressure time durations when the cement pressure was above the thresholds, the differences between positions 1 and 2 were minimal within each pressuriser group. However, with the modified pressuriser these durations were significantly longer than with the conventional design in every pressure point (Two way ANOVA with Bonferroni/Dunn test, p < 0.0005, Figure 4).

**Figure 5:**
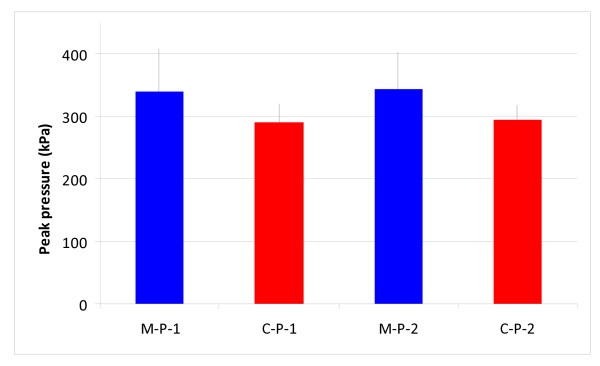
**The peak pressures for positions 1 and 2 for the modified (blue bars) and conventional (red bars) pressuriser**. M-P-1 = Modified position 1 - Proximal. M-P-2 = Modified position 2 - Distal. C-P-1 = Conventional position 1 - Proximal. C-P-2 = Conventional position 2 - Distal

The mean peak pressure at position 1 reached 339.8 ± 68.1 kPa and 290.8 ± 29.6 kPa, and at position 2 reached 343 ± 59.6 kPa and 294 ± 23.7 kPa for the modified and conventional designs, respectively (Figure 4). For each pressuriser design group the difference in cement pressure between positions 1 and 2 was minimal (Modified group p = 0.93; Conventional group p = 0.86). Comparing the new modified with the conventional pressuriser, an increase of 17% was seen for the new design, this difference was, however, not statistically significant (position 1, p = 0.18 and position 2 p = 0.12, Figure 5).

## Discussion

The modified pressuriser generates longer high pressure durations in the femoral model. The pressure was homogenous within the femoral cavity for both pressurisers. Increases in cement pressurisation lead to increased cement penetration [6,7,11-16] while constant high pressure benefits high viscosity cement penetration whether in a constrained cavity such as the femur [17] or a more open area such as the acetabulum [18].

The conventional pressuriser tested here can reach a high pressure, nearly 300 kPa (2256 mmHg), but due to cement leakage did not retain this pressure. The changes of pressures depend of how the pressurisers close the proximal calcar part. The longer taper and smaller circumference of the modified design allows it to sink 5 to 6 mm deeper into the proximal femur resulting in an improved seal with the irregular surface of the resected femoral neck. With more controlled and reduced cement leakage the pressure drops more gradually allowing the high pressure to be retained for longer. The time with high cementation pressure (250 kPa) was about twice that in the modified pressuriser group, whereas in the lower pressure thresholds (100-150 kPa) they were increased by about one third (Figure 4). In an FEA model of cement penetration into the proximal femur it was shown that increasing the pressure by 25% or 50% increased the cement volume in the femur by 17% or 40% respectively, while increasing the pressurisation time by 27% increased the cement volume by 7.5%. It may be that as the modified pressuriser sits slightly deeper in the proximal femur it will cover a small region of the cancellous bone in the most proximal part of the femur, a concern we believe is negligible compared to the better controlled pressurisation. Another risk when increasing the femoral cementation pressure is increased risk of fat and bone marrow emboli entering the blood stream. This consideration emphasizes that when using cement pressurisation it is mandatory to use careful pulse lavage of the femoral canal and thoroughly remove marrow debris prior to the insertion of the cement. As always the patient should be monitored during cement insertion, pressurisation and stem insertion.

Although Sawbones are not identical to real bones they have the advantage of the same basic geometry and therefore the relative pressure distribution should be the same even if there are minor changes produced by flow in to the trabecular space. The study shows that this new design of pressuriser works well in the standardized Sawbone^® ^model. The new pressuriser has, however, also been used clinically in further trials with so far full satisfaction, even if the effect of longer higher pressure durations is not proven for all possible variations of femurs.

With both pressurisers a newly developed metal backing plate (Figure 2) was used to support the pressuriser. We believe that the use of the metal backing plate rather than the surgeon's fingers to support the pressuriser makes the cementation procedure easier and provides a better pressure transfer from the cement gun to the pressuriser, something that is probably true for all silicone pressuriser designs.

## Conclusions

It is possible to achieve a more controlled and longer lasting high cementation pressure in femur with a pressuriser that better seals the femoral opening. This design modification may enhance cement penetration into cancellous bone and make it easier to achieve radiographical "whitening out" when cementing a proximal femoral component.

## Competing interests

The authors declare that they have no competing interests.

## Authors' contributions

JSW, GF and GG designed the study, performed the experiment, did the data analysis and wrote the manuscript. KET was involved in study design and writing the manuscript. FK took part in the performance of the experiment. All authors read and approved the final manuscript.
